# A Combination of Gestalt Therapy, Rosen Body Work, and Cranio Sacral Therapy did not help in Chronic Whiplash-Associated Disorders (WAD) - Results of a Randomized Clinical Trial

**DOI:** 10.1100/tsw.2004.132

**Published:** 2004-12-10

**Authors:** Søren Ventegodt, Joav Merrick, Niels J. Andersen, Tom Bendix

**Affiliations:** ^1^ The Quality of Life Research Center, Teglgårdstræde 4-8, DK-1452 Copenhagen K, Denmark; ^2^ National Institute of Child Health and Human Development, Office of the Medical Director, Division for Mental Retardation, Ministry of Social Affairs, Jerusalem and Zusman Child Development Center, Division of Pediatrics, Ben Gurion University, Beer-Sheva, Israel; ^3^ Norwegian School of Management, Sandvika, Norway and the Scandinavian Foundation for Holistic Medicine, Sandvika, Norway; ^4^ The Back Research Center, Funen Hospital Ringe, University of Southern Denmark, Odense, Denmark

**Keywords:** quality of life, QOL, neck pain, screening, questionnaires, SEQOL, QOL5, whiplash-associated disorders, WAD, human development, alternative and complementary medicine (CAM), holistic medicine, public health, square curve paradigm, life mission theory, Rosen body work, gestalt therapy, Cranio Sacral therapy, Randomized Controlled Trial, RCT, Denmark

## Abstract

The chronic state of whiplash-associated disorder (WAD) might be understood as a somatization of existential pain. Intervention aimed to improve quality of life (QOL) seemed to be a solution for such situations. The basic idea behind the intervention was holistic, restoring quality of life and relationship with self, in order to diminish tension in the locomotion system, especially the neck. A psychosomatic theory for WAD is proposed. Our treatment was a short 2-day course with teachings in philosophy of life, followed by 6 to 10 individual sessions in gestalt psychotherapy and body therapy (Rosen therapy and Cranio Sacral therapy), followed by a 1-day course approximately 2 months later, closing the intervention. Two independent institutions did the intervention and the assessments. In a randomized, clinically controlled setting, 87 chronic WAD patients were included with a median duration of 37 months from their whiplash accidents. One patient never started. Forty-three had the above intervention (female/male = 36/7, ages 22—49, median 37 years) and another 43 were assigned to a nontreated control group (female/male = 35/8, ages 1848, median 38). Six had disability pension and 27 had pending medicolegal issues in each group. Effect variables were pain in neck, arm, and/or head; measures of quality of life and daily activities; as well as general physical or mental health. Wilcoxon test for between-groups comparisons with intention-to-treat analyses was conducted; the square curve paradigm testing for immediate improvements of health and quality of life was also used. The groups were comparable at baseline. From the intervention group, 11 dropped out during the intervention (4 of those later joined the follow-up investigation), 22 of the remaining 32 graduated the course, and 35 of the 43 controls did as well. Approximately 3 months later, we found no clinically relevant or significant increase in any effect measure. The above version of a quality of life intervention based on alternative therapy had no effect on patients with chronic WAD.

### INTRODUCTION

For some years, there has been an intense debate on the nature of whiplash-associated disorders (WAD). The Quebec Task Force Classification has defined five groups for the acute phase of the disease[1]. Most whiplash patients heal spontaneously, but some continue into a chronic phase, reported by different researchers from 2–34%[1,2,3]. In many western countries, 5–20% of the patients still have symptoms 6 months after their injury. Part of the reason for the above variation is the difference in definition, but the fact is that some of the patients will have permanent invalidism and never be able to work again or lead a normal life. This fraction of WAD patients was our target group for intervention.

#### The Biological Damage

In the acute phase, immediately after the physical impact and the whip-like movement of the spine, there is agreement that there might be some physical damage to the muscles, bones, joints, tendons, fascia, and/or the connective tissue, even in the case of no obvious fracture or other gross damage. The issue of discussion is why these often small and dispersed damages do not heal. One theory is that patients with a high quality of life (QOL) worry less about their physical pain, resulting in better healing[5]. One study has demonstrated a number of minor damages, like small cracks in the bones[6], but most MRI studies have not shown any sign of significant damage[7] in the initial phase, except for one study that found minor damages to the disc[8].

The chronic phase has been difficult to understand. One group has worked with the theory that the facet joints are causing the pain and found that about half of the patients have a positive effect from local anesthesia[9,10]. Other groups have found small diversities from normal in rotation pattern[11] or eye movements[12,13], but it has been very difficult to establish a single well-defined organic damage to blame for the diversity of WAD symptoms found. Our group of patients had the typical cocktail of symptoms: pains; difficulties in moving the head, neck, and back; vertigo; low personal energy; lack of concentration; lack of motivation; sleeping disturbances; lack of sexual interest or energy; and a general feeling of being sick.

#### The Psychological and Existential (Deep Psychological) Dimensions

It is believed that psychological factors are of major importance in WAD. One study indicated that the most important predictor for pain and discomfort at a later stage was the patient’s insurance situation, because if the patient was going to fight for insurance money, it was very likely that he or she would have WAD symptoms at a later stage[14]. In countries with less insurance, the average healing time is much shorter[15,16,17]. There has been critique of the conclusions, because the number of participants was small[18] and because of difficulties in comparing the technical circumstances of the incidents[19]. Other indicators of the importance of psychological factors in WAD are the fact that the threshold for sensing pain is dramatically increased with growing mental involvement[4,20]. We all know the extreme situations, such as when you are relaxing at the beach and an ant bite feels like a lion bite or, on the other extreme, when a soldier at war continues to fight in spite of being wounded. Even the *memory* of an old wound, now healed, can cause pain[21,22]. The whole area of psychosomatics is well described in medical science, as are many psychoimmunological factors. A poststress syndrome is found to be a prognostic factor[23]. From the present knowledge, we are forced to conclude that the etiology of WAD is still not well understood and an important contribution from psychological and existential factors must be expected.

Since 1990, the Quality of Life Research Centre in Copenhagen has studied the connection between quality of life and a large number of factors, one among them the sensation of pain[24,25]. In our work with quality of life, we have focused on measuring global and generic quality of life using SEQOL and QOL5, two self-administered, theory-based questionnaires[26,27] and through the Copenhagen Perinatal Birth Cohort 1959–61 follow-up, we looked at long-term aspects of quality of life compared with events in pregnancy and early childhood[25,28].

We believe there is a connection between quality of life and health in the way that the connection is hypothesized to be causal from quality of life to health[29] or, in other words, health improves when quality of life improves. We also believe that this hypothesis (that quality of life and self-perceived health are susceptible dimensions that can be improved considerably in a short time) must be scientifically tested through intervention studies.

### Treatments for WAD

In the acute phase, there are several methods in use from immobilization[30], “live as usual” programs[31], mobilizations[32], and exercise[33], but is not clear if any of these has any preventative effect for the later development of a chronic phase of WAD. The best advice in the acute phase at the moment seems to be to live as usual. For the chronic phase of WAD, there is no cure yet.

### The Philosophy Behind the Study

According to the life mission theory[29], global quality of life is improved when the patient lets go of negative beliefs. According to the theory, negative beliefs are anchored in emotional pain, which is “deposited” in body and mind, giving poor quality of life, poor mental and physical health, and a low ability to function. For practical reasons, the philosophy behind this study was somewhat simplified. The human being is described here as existing in three different inner worlds, which are parallel to each other: a mental world, an emotional world, and a physical world. The reason for compartmentalizing human existence into these three worlds (the mind, feelings, and the body) was that these different worlds seemed to live their own lives inside us to such a degree that they are normally taken as free to develop independently of each other. Training and teaching (for example, in philosophy) can improve our understanding of life. When processing our personal history, therapy can help us towards a healthier emotional life. When we work with our body, we become more present in it, less shameful, for example, about our sexuality, and we can come to feel that “the energies flow”. Such subjective experiences seem to be highly important for the cure for WAD, but most important is the experience of healing our existence, of finally being oneself or the person one was meant to be[29]. It seems that combining work with the body, the feelings, and the mind can give the patient this unique and important experience, which often seems to give the patients an improvement, but we still need to demonstrate that it can be done under controlled circumstances.

### A Psychosomatic Theory for WAD

According to the life mission theory, a psychosomatic theory for WAD would be that our existential pain can take form as blockages in the body. A common form of blockage is the chronic tension of muscles. A person with severe existential pain suppressed in the body can suddenly get sick if these blockages relocate to a sensitive part of the body, such as the neck. After a whiplash lesion, the natural process of healing includes the fixation of all the muscles in the area to protect the tissue while healing. The chronic tension of the muscles in the neck region “invites” the relocation of the blockages to the neck region, where they are much more harmful than their original place, i.e., in the long muscles of the back. Psychologically, there is a great advantage in holding the neck tension as emotional numbness. A person that keeps a lot of tension in the neck is much less emotional than a relaxed person.

The neurotic benefit of the tension in the neck area makes it hard to let go and so, therefore, the WAD. It is very difficult for the patient to reverse the WAD, because this demands that the person take responsibility for his or her own emotions. The cure is to make the person conscious of his of her present emotional state and allow the person to process the hidden existential pain. Our work with the patients and their strong reactions to our attempts to mirror them in their emotional present state (numbness and hidden pain) confirmed this presumption to us.

The purpose of the experiment was to test qualitatively and quantitatively the hypothesis that quality of life and subsequent subjective health could be improved effectively by a combination of philosophy of life, psychotherapy, and body therapy, supporting the salutogenetic process[29,34,35,36].

The factors that were subject to influence were brought together in a concept for the intensive 5-day quality-of-life and health course (5D-QOL intervention)[24]. In the present study, the focus was on using the best from alternative therapy in a theoretically optimal combination of alternative therapy: the combination of gestalt therapy, Rosen Body Work, and Cranio Sacral therapy, especially adapted to the needs of every patient.

The effect of the alternative treatment was optimized by teaching the patient the philosophy of life and health needed for maximal cooperation with the treatments. The concept in the present study was “2 days of philosophical training, followed by 6–10 individual sessions of alternative therapy, and a 1-day follow-up”. The purpose of the experiment was to test qualitatively and quantitatively the hypothesis that quality of life and subsequent subjective health could be improved effectively by a combination of philosophy of life, psychotherapy, and body therapy, supporting the salutogenetic process[34,35]. Unfortunately, the patients were not sufficiently informed about the alternative treatment, bringing their existential problems up to the surface of consciousness, which can be emotionally painful, so many patients resisted the alternative treatment.

### METHODS

This study had a prospective, randomized, controlled design, where an intervention group was compared to a nontreated control group. The patients were recruited through their family physician or from newspaper advertisements. The treatment was a simple intervention with alternative medicine and philosophy of life according to the protocol.

#### Criteria of Inclusion

Hit in a car from behind by another car in an angle of between +45 and –45 degrees. Time since accident had to be from 6 months to 10 years. Average pain level had to be at least 5 on a visual box scale from 0 to 10 and age 18–70 years.

### Criteria of Exclusion

- Signs of fracture or dislocation (Quebec Task Force category 4)
- Mental disease, such as depression and other severe psychiatric disease
- Disease making interpretations of symptoms difficult
- Significant problems with neck prior to whiplash
- Abuse of alcohol or medicine

### Baseline Examination

- Objective examination: Palpation of eight preselected muscles; Cervical Range of Motion by CROM instrument.
- SPNT-test: Smooth Persuit Neck Torsion test as described in [12,13]
- Questionnaire on:
  - Symptoms
  - Time for inset of symptoms
  - Development of symptoms since the inset
  - Intensity of pain
  - Body area hit by pain
  - Paraesthesia
  - Symptoms that are not painful (vertigo, disturbance of vision, tiredness, irritability, anxiety, memory-difficulties, difficulties sleeping, and hypersensitivity to noise)
  - Technicalities about the accident
  - Social data
  - Global quality of life (SEQOL)

The following concept for measuring global, generic quality of life was used[26]:

1. A clear definition of the quality of life
2. A philosophy of life on which the definition of quality of life was based
3. A theory that makes this philosophy operational by deducing questions that are unambiguous, mutually exclusive, and comprehensive as a whole and establishing the relative weights of each question
4. A number of response options that can be quantitatively interpreted on a fraction scale
5. Technical quality in terms of reproducibility, sensitivity, and “well scaledness” (appropriate scale characteristics)
6. The survey must be meaningful to researchers, respondents, and those who use the results (including criterion validity)
7. An appreciation of the aesthetic dimension

Immediate subjective well being was measured with only one question on a five-point symmetric Likert scale, as were satisfaction with life and happiness. Satisfaction of needs was measured with five questions according to a modified theory of needs based on Maslow’s hierarchy of needs. All five questions were rated on five-point Likert scales. The composite global quality-of-life measure “family, work, and leisure time” rated global quality of life at home, at work, and in the leisure time using three questions and three five-point Likert scales. The “quality of relationships” was given an average of the rating of all close relationships on five-point Likert scales. Total quality of life was calculated as usual in SEQOL that is according to the integrated quality-of-life theory (IQOL theory), except that objective factors were not included in this study[26].

### Intervention

The 3 days of training in philosophy of life had the purpose of giving the patients a positive perspective and basic understanding of life[5,24,29,36]. The factors that were subject to influence were brought together in a concept for the intensive 5-day quality-of-life and health course (5D-QOL intervention)[24,38]. In the present study, the focus was on using the best from alternative therapy in a theoretically optimal combination of alternative therapy: the combination of gestalt therapy, Rosen Body Work, and Cranio Sacral therapy, especially adapted to the needs of every patient.

The effect of the alternative treatment was optimized by teaching the patient the philosophy of life and health needed for maximal cooperation with the treatments. The concept in the present study was “2 days of philosophical training, followed by 6–10 individual sessions of alternative therapy, and a 1-day follow-up”.

### Outcome Measures

At 3 months after the last session, the participants received a questionnaire where outcome measures were pain level, daily functioning, and ability to work/have work, as well as an overall measure of quality of life.

### Stratification and Statistical Methods

The patients were placed in the two groups using a PC program[39]. The analyses were made as nonparametric comparisons of two independent groups (the Mann-Whitney and $X^2$ test).

### Ethical Considerations

There is no known cure for the WAD patients included. The quality-of-life intervention is believed not to have negative side effects. The Danish Scientific Ethical Committee (IRB) accepted the project.

## THE SQUARE CURVE PARADIGM

The square curve paradigm is a simple way to test the efficiency (clinical significance) of a cure that improves the quality of life health within a month[40]. As we believed that an efficient cure for WAD would have measurable results within this time frame, we hoped to be able to document the efficiency of the alternative treatment, but we failed to see this improvement. The name of the paradigm comes from the “square curve” made of a base line, a sudden vertical jump during a successful intervention, and a new baseline (a constant level of quality of life, health, and/or ability) on a significantly higher level (Fig. 1).

## RESULTS

Unfortunately, the study was not fulfilled as planned. The treatment schedule became somewhat irregular and the motivation was generally poor, in part due to a public criticism of the concept. The project leader declined from fulfilling the study due to this.

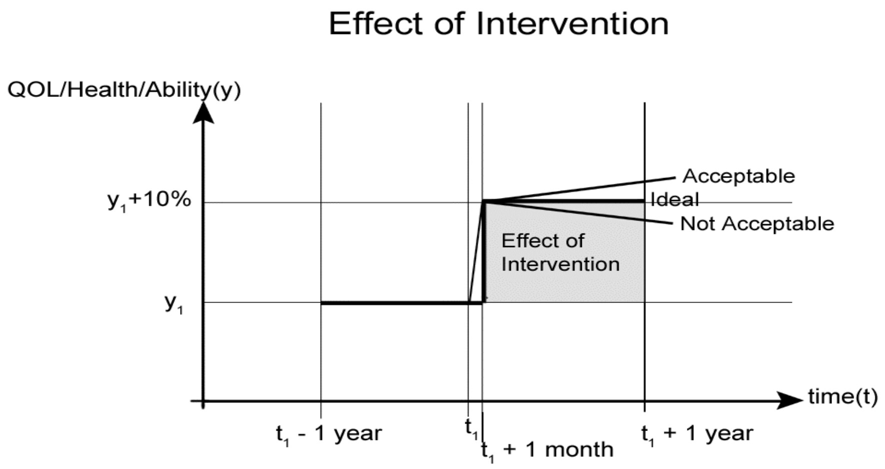

**FIGURE 1.** The "square" curve paradigm; the ideal curve that documents scientifically valid and clinically relevant effect of alternative and holistic medicine is squared, since the group, on average, is on one level before and a higher level after the treatment. The treatment intervention must be of limited duration. Endpoints are quality of life, health, and/or the ability to function. The area under the square curve is the documented gain of the patients. For the square curve to appear, time must be measured at *T1 minus one year* (sufficiently long time before the intervention), at *T1* (immediately before the intervention), at *T1 plus one month* (immediately after the intervention), and at *T1 plus one year* (sufficiently long time after the intervention). If the patients are chronically ill and the situation is stabile, the measurement *T1 minus one year* is not necessary. Please note that if the effect is lost through time, which is when the difference between Y(*T1 plus one year) and* Y(*T1 plus one month)* is negative and larger that the insecurity of the measurement, the effect is not documented (the curve marked "not acceptable").

The plan was to include 120 patients, but only 87 patients were randomized with 44 in the intervention group and 43 in the control group. The average time since accident was 37 months. The intervention took place at the Back Research Center at the Funen Hospital Ringe on the 18–19$^{th}$ of May and the 7–8$^{th}$ of September 2002. The 1-day follow-up took place on the 11$^{th}$ of January 2003. Questionnaires were sent to the participants on the 14$^{th}$ of April (3-month follow-up). One patient dropped out before the beginning, 32 completed the intervention, while another 11 dropped out during the treatment. The main reason for this drop out was that the participants found the intervention and the therapists too provoking. Of the 32 completing the intervention, 22 filled out the questionnaires at 3 months. Four of the patients who dropped out and 35 of the controls also completed the questionnaires (see Fig. 2).

In Table 1, results from the intervention and control group are shown at the time before the intervention, while Table 2 shows the data 3 months after the terminated intervention on the two measurements of quality of life, health, and the above-mentioned WAD-related dimensions.

We found no significant difference between the intervention group and the control ($p$ = 0.28) on global quality of life or any of the health dimensions.

### DISCUSSION

Scarcity of resources, a general belief in holistic therapy, and trust in some of the most famous and well-known alternative therapists in Denmark, made us change the formerly seemingly successful 5-day intervention to "2 days of philosophy of life, 6–10 individual sessions of alternative therapy, and 1 day of follow-up", which may be one reason for the lack of demonstrated effect.

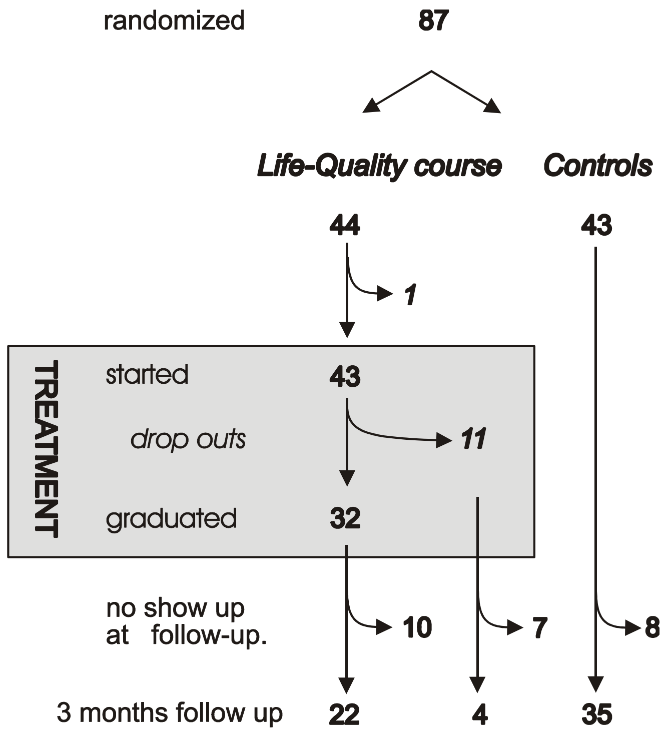

**FIGURE 2.** The patient flow through the study.

We succeeded in recruiting some of the most recognized spine therapists in Denmark, several of them having 20 years of experience and running their own therapeutic training institute. The justification for this modification was the belief that individual sessions of Rosen therapy, Cranio-Sacral therapy, and gestalt therapy supporting the different steps of the holistic healing would have a strong beneficial effect on the WAD patients. We have learned from this study that our assumption was false with this patient group.

At the motivation of the patient to help himself believed to be a key factor in alternative medicine, a next study with alternative medicine must use the dimension of motivation as an inclusion criteria.

Patients were informed that the project was going to offer them a new experimental treatment for WAD; the emphasis was not sufficiently laid on the dimension of personal development. When the subjects arrived for the 2-day course, they were not motivated for personal development, as they did not expect that as the central core of the project. At a time during the 2 days, it was obvious that one-third of the patients had made an alliance to resist the fairly painful holistic process that was the essence of the possible rehabilitation. These patients refused to cooperate with the therapists and caused a lot of disturbances. The emotional pain, according to the theory that was the hidden etiology behind the WAD, was easily seen and surfaced readily, but many of the patients could not take responsibility for it and blamed the therapist for their pain.

In the therapeutic team, we had to conclude that we failed to get the patients into a holistic process of healing[41,42]. In the pilot studies, we managed to include highly motivated patients. It was the impression of the treatment staff that in this study it turned out quite opposite, since we recruited patients without much motivation for personal development. To make it work better, it is of crucial importance that the patients know exactly what they are signing up for, which therapist and methods they will meet, what is expected from them, and which concepts, ideas, and philosophy they will find in the project.

**Table 1**

**Intervention Group vs. Control Group (Initial Values Before Intervention)**

| | | N = | Median/Ratio | Range | IQR | p (Intervention = Control Group ?) |
|---|---|---|---|---|---|---|
| Gender | All in intervention group | 43 | F:36 M:7 | | | 0.78 |
| | graduating | 32 | F:27 M:5 | | | 0.74 |
| | Control group | 43 | F:35 M:8 | | | |
| Age | All in intervention group | 43 | 37 | 22–49 | 33–43 | 0.79 |
| | graduating | 32 | 38 | 23–49 | 33–44 | 0.72 |
| | Control group | 43 | 38 | 18–48 | 31–44 | |
| Sick leave/WL | All in intervention group | 40 | Y:10 N:30 | | | 0.85 |
| | graduating | 31 | Y:6 N:25 | | | 0.46 |
| | Control group | 41 | Y:11 N:30 | | | |
| Pension/WL | All in intervention group | 43 | Y:6 N:37 | | | 1.0 |
| | graduating | 32 | Y: 5 N:27 | | | 0.84 |
| | Control group | 43 | Y:6 N:37 | | | |
| Medicolegal issues | All in intervention group | 43 | Y:27 N:16 | | | 1.0 |
| | graduating | 32 | Y:20 N:12 | | | 0.98 |
| | Control group | 43 | Y:27 N: 16 | | | |
| Neck pain | All in intervention group | 43 | 7 | 2–10 | 5–8 | 0.14 |
| | graduating | 32 | 7 | 2–10 | 5–8 | 0.24 |
| | Control group | 43 | 6 | 0–10 | 4–8 | |
| Arm pain | graduating | 43 | 4 | 0–10 | 1–5 | 0.75 |
| | graduating | 32 | 3 | 0–10 | 1–5 | 0.69 |
| | Control group | 41 | 3 | 0–10 | 0.5–5.5 | |
| Headache | All in intervention group | 43 | 7 | 1–10 | 5–8 | 0.32 |
| | graduating | 32 | 7 | 1–10 | 5–8 | 0.30 |
| | Control group | 43 | 6 | 0–10 | 4–8 | |
| Global QOL | All in intervention group | 43 | 3 | 1–5 | 2–3 | 0.14 |
| | graduating | 32 | 2.5 | 1–5 | 2–3 | 0.42 |
| | Control group | 43 | 2 | 1–5 | 2–3 | |
| Health, physical | All in intervention group | 43 | 3 | 1–5 | 2–4 | 0.53 |
| | graduating | 32 | 3 | 1–5 | 2–4 | 0.88 |
| | Control group | 43 | 3 | 1–5 | 2–4 | |
| Health, mental | All in intervention group | 42 | 2 | 1–5 | 2–3 | 0.61 |
| | graduating | 31 | 2 | 1–5 | 2–3 | 0.77 |
| | Control group | 43 | 2 | 1–5 | 2–3 | |
| Revalidation | All in intervention group | 40 | Y:2 N:38 | | | 0.69 |
| | graduating | 31 | Y:1 N:30 | | | 0.47 |
| | Control group | 42 | Y:3 N:39 | | | |

N = number of participants, F = female, M = male, Y = yes, N = no, IQR = interquartile range, p = level of significance. Differences between the numbers in Fig. 2 and that of the Table reflect missing values.

**TABLE 2**
**Intervention Group vs. Control Group (3 Months after Intervention)**

| | | N = | Median | Range/Ratio | IQR | p (Intervention = Control Group ?) |
|---|---|---|---|---|---|---|
| Neck pain | All in intervention group | 26 | 6 | 1–8 | 4–7 | 0.86 |
| | graduating | 22 | 6 | 1–8 | 4–7 | 0.56 |
| | Control group | 34 | 6 | 1–9 | 5–8 | |
| Arm pain | All in intervention group | 26 | 4 | 0–9 | 1–6 | 0.94 |
| | graduating | 22 | 4 | 0–9 | 1–6 | 0.68 |
| | Control group | 34 | 4 | 0–9 | 4–9 | |
| Headache | All in intervention group | 26 | 5.5 | 1–9 | 4–7 | 0.79 |
| | graduating | 22 | 5 | 1–9 | 3–6 | 0.69 |
| | Control group | 34 | 5 | 1–10 | 4–7 | |
| Sick leave/WL | All in intervention group | 26 | Y:8 N:18 | | | 0.19 |
| | graduating | 22 | Y:6 N:16 | | | 0.30 |
| | Control group | 29 | Y:4 N:25 | | | |
| Sick leave/not WL | All in intervention group | 28 | Y:1 N:27 | | | 0.10 |
| | graduating | 21 | Y:0 N:21 | | | |
| | Control group | 29 | Y:1 N:28 | | | |
| Global QOL | All in intervention group | 26 | 3 | 2–4 | 2–3 | 0.28 |
| | graduating | 22 | 3 | 2–4 | 2–3 | 0.24 |
| | Control group | 35 | 3 | 1–5 | 3–3 | |
| ADL, sum | All in intervention group | 26 | 16.5 | 1–25 | 13–20 | 0.57 |
| | graduating | 20 | 15.5 | 1–25 | 13–20 | |
| | Control group | 33 | 15 | 0–27 | 9–21 | |
| Sleep | All in intervention group | 25 | 2 | 1–4 | 2–3 | 0.43 |
| | graduating | 22 | 2 | 1–4 | 2–3.3 | 0.58 |
| | Control group | 30 | 2 | 1–4 | 1–3 | |
| Vertigo | All in intervention group | 23 | 2 | 1–4 | 2–3 | 0.73 |
| | graduating | 20 | 2 | 1–4 | 2–3 | 0.99 |
| | Control group | 29 | 2 | 1–3 | 1.5–3 | |
| Health, physical | All in intervention group | 26 | 3 | 1–5 | 2–4 | 0.40 |
| | graduating | 22 | 3 | 1–4 | 2–3.3 | 0.24 |
| | Control group | 34 | 3 | 1–5 | 2–4 | |
| Health, mental | All in intervention group | 26 | 2 | 1–5 | 1.8–3 | 0.18 |
| | graduating | 22 | 2 | 1–5 | 1–3 | 0.16 |
| | Control group | 33 | 2 | 1–5 | 2–3.5 | |
| Pension/WL | All in intervention group | 26 | Y:4 N:22 | | | 0.91 |
| | graduating | 22 | Y:3 N:19 | | | 0.95 |
| | Control group | 25 | Y:5 N:20 | | | |
| Revalidation | All in intervention group | 11 | Y:3 N:8 | | | 0.94 |
| | graduating | 9 | Y:2 N:7 | | | 0.72 |
| | Control group | 21 | Y:6 N:15 | | | |

N = number of participants, F = female, M = male, Y = yes, N = no, IQR = interquartile range, p = level of significance. Differences between the numbers in Fig. 2 and that of the Table reflect missing values.

It is very important to mention, amidst the obvious failure, that we saw half the patients becoming better for short periods of time. Three of the patients were completely free from any symptoms for hours and days, and several of them declared at the end of the study that they felt their quality of life had improved and the impact of the WAD had diminished radically. However, due to the overall equality in effect, the treatment must have worked in the opposite direction for some other patients.

At the end of the intervention, we talked with the patients about their subjective experience of their health and quality of life. Fifteen patients said their WAD symptoms were the same as before, two said they felt worse than before, and five said the symptoms had diminished. Five patients said that their quality of life was the same, 3 felt worse than before, and 15 said they felt better in some way or another than before entering the study. One patient openly expressed the frustration over "falling down again" from a period of 8 h completely free of any WAD symptoms after years of constant suffering.

What we learned was that the complex psychobiological scene inside the WAD patient was obviously highly dynamic, and in this way WAD looked like a psychosomatic disease. The opinion of the treatment staff was that the observed dynamics may be explained like this: A very important understanding in holistic medicine is that problems can be moved from the physical body to the emotional sphere, but if they are not confronted and processed here, they will return to the physical body and the symptoms will reappear. The patient can only become healthier and feel better if he understands the problem well enough to find and let go of the negative decision that ties his life to the "ground". It is the essence of the life mission theory, the basis of the philosophy behind this study. This could be an explanation for the lack of an observed effect that the patients may not have achieved this understanding.

### CONCLUSIONS

A combination of a weekend course with quality-of-life (QOL) philosophy and 6–10 sessions of alternative therapy (Rosen Body Work, gestalt therapy and Cranio Sacral therapy) with a treatment series fitted for every patient's personal needs, could not improve the quality of life and health status for WAD patients in the actual setting. So we must conclude that: "*As regretful it is for man to fail, so fruitful it is for him to learn from his failure.*"

### ACKNOWLEDGMENTS

The Danish Scientific Ethical Committee (IRB) accepted the project. The study was primarily supported by grants from IMK Almene Fond. It was based on general Quality of Life Survey, which has been granted from: Apoteker-fonden af 1991, De praktiserende lægers Goodwill-fond, JL Fondet, Direktør E. Danielsen og Hustrus Fond, Emmerick Meyers legat, Frimodt-Heineken fonden, Familien Hede Nielsens Fond, Petrus Andersens Fond, Grosserer C.P. Frederiksens Studielegat for medicinske studenter og unge læger and Else & Mogens Wedell-Wedellsborgs Fond, IMK Almene Fond, and others.

### BIOSKETCHES

**Søren Ventegodt, MD,** is the Director of the Quality of Life Research Center in Copenhagen, Denmark. He is also responsible for a Research Clinic for Holistic Medicine in Copenhagen and is a popular speaker throughout Scandinavia. He has published numerous scientific or popular articles and a number of books on holistic medicine, quality of life, and quality of working life. His most important scientific contributions are the comprehensive SEQOL questionnaire, the very short QoL5 questionnaire, the integrated QOL theory, the holistic process theory of healing, the life mission theory, and the Danish Quality of Life Research Survey, 1991–94 in cooperation with the University Hospital of Copenhagen and the late pediatric professor Bengt Zachau-Christiansen. E-mail: ventegodt@livskvalitet.org. Website: http://www.livskvalitet.org

**Joav Merrick, MD, DMSc,** is Professor of Child Health and Human Development affiliated with the Zusman Child Development Center, Division of Pediatrics and Community Health at the Ben Gurion University, Beer-Sheva, Israel; the Medical Director of the Division for Mental Retardation, Ministry of Social Affairs, Jerusalem; and the Founder and Director of the National Institute of Child Health and Human Development. He has numerous publications in the field of child and human development, rehabilitation, intellectual disability, disability, health, welfare, abuse, advocacy, quality of life, and prevention. Dr. Merrick received the Peter Sabroe Child Award for outstanding work on behalf of Danish Children in 1985 and the International LEGO-Prize ("The Children's Nobel Prize") for an extraordinary contribution towards improvement in child welfare and well being in 1987. E-mail: jmerrick@internet-zahav.net. Website: www.nichd-israel.com

**Niels Jørgen Andersen, MSc,** Professor, Department of Innovation and Economic Organization, Norwegian School of Management. This department conducts research and provides teaching in central topics related to innovation, business development, management of global companies, business history, and economic organization. Research activities within the department are related to four core subjects within the discipline: business history, cooperative organizations, business development and entrepreneurship, and finally studies of industries with a special focus on the electricity industry. He is also the dynamic chairman of the nonprofit organization Stiftelsen Holistisk Medisin Scandinavia, that aims to support the scientific development, research, and documentation of complementary and holistic medicine in Scandinavia. E-mail: niels.j.andersen@bi.no. Website: www.bi.no/users/fgl93013/

**Tom Bendix**, **MD, PhD**, Professor at the Institute of Sports Medicine and Clinical Biomechanics, University of Southern Denmark, Odense. Also head of research at The Back Research Center, Funen Hospital Ringe. E-mail: tbendix@health.sdu.dk. Website: http://www.sdu.dk/health/iob/ansatte/tb.htm
